# How we see the forest and the trees

**DOI:** 10.7554/eLife.41633

**Published:** 2018-10-12

**Authors:** Jeffrey S Diamond

**Affiliations:** Synaptic Physiology SectionNational Institute of Neurological Disorders and StrokeBethesdaUnited States

**Keywords:** nonhuman primate, natural scenes, receptive field, retinal ganglion cell, vision, neurotransmitters, Rhesus macaque, None

## Abstract

Signaling pathways in the retina help us see spatial detail in our visual world.

**Related research article** Turner MH, Schwartz GW, Rieke F. 2018. Receptive field center-surround interactions mediate context-dependent spatial contrast encoding in the retina. *eLife* **7**:e38841. doi: 10.7554/eLife.38841

Imagine you are walking through an alpine forest on a beautiful fall day, passing a stand of aspen trees with their thin trunks forming a vertical grid before a brilliant backdrop of autumn color. A closer look reveals the horizontal striations in their white bark (Figure 1A,B). This simple, sylvan example highlights how our visual system seamlessly shifts its attention across the broad range of spatial frequencies in the natural world: it can report global shapes, patterns and motion, and also encode fine details, enabling us to see the forest – and the trees.

**Figure 1.**
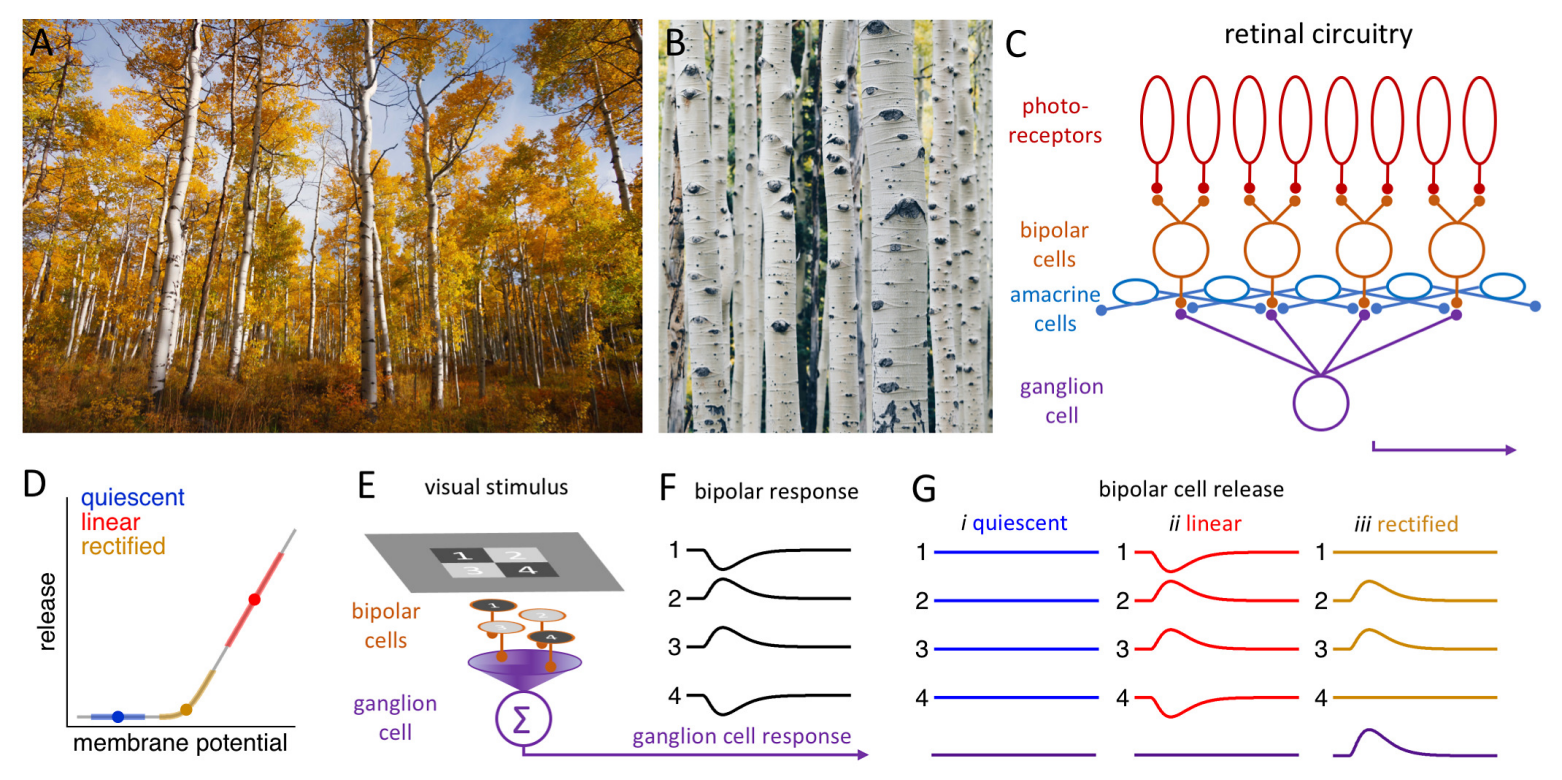
Processing visual information in the retina. (**A**) A stand of aspen trees, seen from a distance, presents primarily vertical lines (Image credit: John Price). (**B**) Closer inspection reveals primarily horizontal features in the bark of individual trees (Image credit: Peng Chen). (**C**) Simplified schematic of the retinal circuitry showing the synapses between photoreceptors (top) and bipolar cells, and between bipolar cells and a single ganglion cell. The amacrine cells influence the behavior of the bipolar cells (and the ganglion cells). (**D**) Neurotransmitter release by bipolar cells (y-axis) versus the membrane potential of these cells. Bipolar cells inhabit one of three release regimes: quiescent (blue), when visual stimulation is insufficient to evoke release; linear (red), when release is proportional to the stimulus; and rectified (gold), when only positive stimuli evoke release. (**E**) Schematic showing a checkerboard stimulus presented to a 2 × 2 array of bipolar cells. (**F**) The change in the membrane potential (y-axis) over time (x-axis) of each bipolar cell depends on whether it receives a positive stimulus (2 and 3) or a negative stimulus (1 and 4) from the checkerboard. (**G**) The release of neurotransmitters from the four bipolar cells and the resulting response in the ganglion cell (bottom) depend on the release regime occupied by the bipolar cell (see main text).

One might expect that such a sophisticated system would require this information to be sent to 'higher' visual centers in the brain for processing. However, much of this processing is actually carried out at a relatively 'low' level by the neurons in the retina (Hochstein and Shapley, 1976; Demb et al., 1999; Schwartz et al., 2012; Grimes et al., 2014; Turner and Rieke, 2016). Now, in eLife, Maxwell Turner and Fred Rieke of the University of Washington, and Gregory Schwartz of Northwestern University, report how circuits in the retina fine-tune their spatial sensitivity in response to the surrounding visual world (Turner et al., 2018).

Neurons communicate with each other by releasing signaling molecules called neurotransmitters into the synaptic gaps between them. In the retina, visual signals in the form of slow, graded changes in membrane potential are transmitted from photoreceptors (the cells that actually detect the light we see) to bipolar cells and then to ganglion cells (Figure 1C). The release of neurotransmitters from bipolar cells into a synapse depends on the value of the membrane potential of the neuron relative to the activation range of the calcium ion channels that trigger the release (Figure 1D). There are three different regimes: the 'quiescent' regime, in which only a very strong positive stimulus will evoke release; the 'rectified' regime, in which a positive stimulus will evoke release, but a negative stimulus will not; and the 'linear' regime, in which a positive stimulus will lead to an increase in release, and a negative stimulus will lead to a decrease. Many of the synapses formed by bipolar cells operate in the 'rectified' regime.

Turner et al. studied how visual signals are transmitted from a number of bipolar cells to a single ganglion cell. This transmission depends on which regime the bipolar cells are in, particularly when the intensity of the visual image being transmitted varies across the receptive field of the ganglion cells (Croner and Kaplan, 1995).

Suppose that the bipolar cells in a 2 × 2 array are stimulated independently by a checkerboard image, with two cells receiving a positive stimulus and two receiving a negative stimulus (Figure 1E,F). If the bipolar cells are quiescent, the stimuli will not evoke a release from any of the four cells, and hence no signal will be transmitted to the ganglion cell. Likewise, if the bipolar cells are in the 'linear' regime, the release of neurotransmitters from two of the cells will increase, and the release from two will decrease, thus cancelling each other out, so the signal being transmitted to the ganglion cell will not change. Linear responses can, therefore, diminish the responses of the ganglion cells to higher spatial frequencies. However, if the bipolar cells are in the 'rectified' regime, only the two positively stimulated bipolar cells will release a neurotransmitter, enabling the ganglion cells to respond (Figure 1G; Enroth-Cugell and Robson, 1966).

Another set of cells in the inner retina, the amacrine cells, are also involved regulating the release of neurotransmitters by bipolar cells and thus fine-tuning the information transferred to ganglion cells. In particular, the amacrine cells contribute to the 'center-surround' organization of the receptive fields of ganglion cells: put simply, this means that if a ganglion cell is excited by a stimulus in the center of its receptive field, a similar stimulus in the surrounding area will be inhibitory.

Turner et al. show that ‘surround inhibition’ can influence the spatial sensitivity of the ganglion cells by shifting the bipolar cells from one release regime to another. Strong surround inhibition pushes bipolar cells toward quiescence, limiting responses to center stimuli. Conversely, surround stimuli of the opposite polarity to that of the center decreases inhibition in the surround, pushing the bipolar cells into their linear regime. As a result, contrasting details in the center cancel each other, reducing the ganglion cells’ spatial sensitivity. This proves useful when visual features change abruptly on a larger spatial scale, and encoding global contrast or motion takes temporary precedence over the finer details.

In our spatially correlated natural world, however, the luminance of the center and surround are often similar, so that bipolar cells occupy their rectified regime, thereby maximizing the sensitivity of the ganglion cells to higher spatial frequencies (Field, 1987). Notably, these changes can occur quickly, enabling the circuit to adapt in real time to changing visual conditions.

The work by Turner et al. and others has certainly expanded our appreciation for the remarkable versatility and computational power of this thin, transparent sheet of neurons that lines the back of the eye (Gollisch and Meister, 2010). The retinal circuitry has revealed itself as a dense forest of connected trees that holds many more secrets yet to be discovered.
